# Prevalencia de trastornos temporomandibulares mediante el índice anamnésico simplificado de fonseca en estudiantes de odontología de la universidad juárez del estado de durango, méxico

**DOI:** 10.21142/2523-2754-0902-2021-059

**Published:** 2021-06-21

**Authors:** Luis Javier Solís-Martínez, Víctor Hiram Barajas-Pérez, Óscar Eduardo Almeda-Ojeda, Adán Campuzano-Estrada, Karla Yareli Valles-Flores, Edgar García-Torres

**Affiliations:** 1 Facultad de Odontología, Universidad Juárez del Estado de Durango. Victoria de Durango, México. javier.solis@ujed.mx, hiram.barajas@ujed.mx, oscar.almeda@ujed.mx, adance-17@hotmail.com, karla_yarely93@hotmail.com, smiledent.coi@hotmail.com Facultad de Odontología Universidad Juárez del Estado de Durango Victoria de Durango México javier.solis@ujed.mx hiram.barajas@ujed.mx oscar.almeda@ujed.mx adance-17@hotmail.com karla_yarely93@hotmail.com smiledent.coi@hotmail.com

**Keywords:** trastornos de la articulación temporomandibular, articulación temporomandibular, prevalencia, Temporomandibular joint disorders, temporomandibular joint, Prevalence

## Abstract

**Objetivo.:**

Estimar la prevalencia de trastornos temporomandibulares (TTM) en estudiantes de Odontología de la Universidad Juárez del Estado de Durango, México.

**Material y métodos.:**

Se trata de un estudio descriptivo, observacional, prospectivo y transversal. El universo de estudio contempló a la población estudiantil de la Facultad de Odontología, de la Universidad Juárez del Estado de Durango (México). Se incluyeron alumnos que cursaron del 1.o al 8.o semestre durante el ciclo escolar 2018-A, con edades entre los 18 y 28 años, a quienes se les aplicó un instrumento (índice anamnésico simplificado de Fonseca) que permitió caracterizar los TTM. El tamaño de la muestra se determinó utilizando el software Epi InfoTM y se obtuvo un tamaño de muestra total de 263 individuos. Para describir los datos, se utilizó el paquete estadístico R Studio Team (2019).

**Resultados.:**

La prevalencia total de TTM en la población estudiada fue del 63% y el TTM más prevalente fue el leve, con un 44%. La prueba Ji2 entre el sexo y el TTM muestra significancia estadística (p = 0,001), igual que entre el sexo y los ítems del índice simplificado de Fonseca: ítem 4 (p = 0,001), ítem 7 (p = 0,021), ítem 8 (p = 0,021), ítem 9 (0,001) y el ítem 10 (p = 0,001).

**Conclusiones.:**

Existe una alta prevalencia de TTM en la población estudiantil de la Facultad de Odontología de la Universidad Juárez del Estado de Durango (México), y el sexo femenino tiene una relación con la presencia y la manifestación de síntomas en estos trastornos.

## Abstract (en)

**Objective:**

The objective was to estimate the prevalence of temporomandibular disorders (TMD), in students of the Faculty of Dentistry.

**Material and Methods:**

This was a descriptive, observational, prospective, cross-sectional study. The study included 18 to 28-year-old students from the Faculty of Dentistry of the Universidad Juárez del Estado de Durango, México, attending the 1st to 8th semester of the 2018-A school year in whom the simplified Fonseca anamnestic index (sFAI) was applied to characterize TMD. The sample size was determined using the Epi InfoTM software, obtaining a total sample size of 263 individuals. The R Studio (2019) statistical package was used to describe the data.

**Results:**

The prevalence of TMD in the study population was 63%, with a mild disorder being the most prevalent in 44%. The Chi2 test showed statistically significant differences between sex and TMD (p = 0.001) and between sex and 5 items of the sFAI: item 4 (p= 0.001), item 7 (p= 0.001), item 8 (p = 0.021), item 9 (p= 0.001) and item 10 (p = 0.001).

**Conclusions:**

There is a high prevalence of TMD in the student population of the Faculty of Dentistry of the Universidad Juárez del Estado de Durango, Mexico, with females presenting a higher prevalence of the presence and manifestation of symptoms in these disorders.

## INTRODUCCIÓN

La articulación temporomandibular (ATM) es una articulación ginglimoartroidal, que permite realizar movimientos rotacionales y de desplazamiento; además, orienta y limita los movimientos de la mandíbula, por lo que interviene en la masticación, la deglución, la respiración, el habla y la gesticulación facial ^1^. Entre las disfunciones que pueden afectar la ATM se encuentran los trastornos temporomandibulares (TTM), representados por un conjunto de afecciones que se caracterizan por un movimiento limitante simétrico o asimétrico, y ruido articular acompañado de dolor en zona auricular o en los músculos de la masticación, que incluso puede extenderse al cuello, el oído y la cabeza, lo que corresponde a síndromes disfuncionales de la articulación ^2^^,^^3^.

La prevalencia de los TTM varía entre los individuos y poblaciones, pero de manera general afecta al 25% de la población ^(4, 5)^. En los niños se aproxima al 20%, en adolescentes alcanza el 35%, mientras que en adultos suma un 49% ^6^^-^^9^. La etiología de estos trastornos se considera multifactorial, porque abarca hábitos bucales, ansiedad, estrés, trauma, mal oclusiones, disfunciones articulares y musculares, disfunciones de crecimiento y desarrollo de maxilares, traumatismos y enfermedades como artritis reumatoide ^4^.

Se diagnostica, principalmente, a través de una historia médico clínica y una exploración física; sin embargo, son los estudios de imagen como la radiografía, la artrografía, la ecografía, la resonancia magnética y la tomografía computarizada los que ofrecen un mejor diagnóstico y panorama del daño articular, con la desventaja de ser costosos y utilizados generalmente en casos de dolor extremo y afecciones severas ^10^. Por otra parte, se cuenta con índices de diagnóstico y estudio de TTM que ofrecen la ventaja de que se pueden aplicar en grandes poblaciones en un corto tiempo, para evaluar las diferentes etapas de su manifestación y no solo en casos graves. Entre ellos destaca el índice anamnésico simplificado de Fonseca (IASF), el cual ofrece la ventaja de ser utilizado para estudios epidemiológicos de TTM ^11^.

En la actualidad, no se cuenta con datos sobre la prevalencia de TTM en nuestra población estudiantil considerada adulta, por lo que decidimos implementar el IASF para evaluar de manera no invasiva y sencilla la presencia de TTM en alumnos de la Facultad de Odontología, carrera que se considera muy demandante a nivel físico y mental. El propósito es tener un panorama actual de estos trastornos en nuestra comunidad estudiantil, con el objetivo general de estimar la prevalencia de TTM en estudiantes de Odontología de la Universidad Juárez del Estado de Durango, en México.

## MÉTODOS

Se trata de un estudio descriptivo, observacional, prospectivo y transversal. El universo de estudio contempló a la población estudiantil de la Facultad de Odontología, de la Universidad Juárez del Estado de Durango (México). Se incluyeron alumnos, de manera aleatoria, sin evidencia de enfermedades sistémicas o neuromotoras, que cursaban del 1.^o^ al 8.^o^ semestre, durante el ciclo escolar 2018-A, que decidieron participar en el estudio. Después de firmar una carta de consentimiento informado, se les aplicó un instrumento que permitiera caracterizar los TTM, denominado IASF (versión en español).

### Instrumento de evaluación de TTM

El IASF es un cuestionario conformado por 10 preguntas (ítems) con tres opciones de respuesta: “sí”, con un valor de 10 puntos; “a veces”, con un valor de 5 puntos, y “no”, equivalente a 0 puntos. La puntuación final se determina por la suma de los puntos de cada ítem y se establece la siguiente clasificación: no presenta, leve, moderada y severa. Este IASF fue creado y validado en San Pablo, Brasil, por el Dr. Dickson Fonseca ^12^, para después ser replicado y validado en el idioma español por Jaime A. Lázaro, en Lima, Perú ^13^ (anexo 1).

Se excluyó a los alumnos sometidos a tratamientos de exodoncia, endodoncia, rehabilitación protésica dental o alguna intervención quirúrgica bucal durante los últimos seis meses. Aquellos con un diagnóstico establecido de disfunción temporomandibular tampoco fueron considerados. Se aplicó únicamente el IASF, sin incluir ningún otro examen clínico o toma de muestra. Para el análisis estadístico, se tomaron como variables de interés los ítems del IASF con mayor frecuencia de la respuesta “Sí”. De igual manera, se tomó en cuenta la variable del sexo, representada por 173 mujeres y 90 hombres. 

El tamaño de la muestra se determinó utilizando el *software* de libre distribución Epi Info^TM^ (versión 7.2.1, CDC Atlanta, EE. UU.), a partir de los siguientes supuestos: encuesta descriptiva mediante muestreo aleatorio N = 560, frecuencia esperada de TTM del 33,8%, límite de confianza del 5%, y se obtuvo un tamaño de muestra de 263 individuos. La frecuencia esperada para calcular el tamaño de muestra se tomó de estudios anteriores en población mexicana ^9^. Se utilizaron medidas de tendencia central y dispersión, porcentajes y análisis de independencia para describir los datos, todo esto utilizando el paquete estadístico R Studio, de libre distribución ^14^.

Las consideraciones éticas se siguieron de acuerdo con la ley general de salud de los Estados Unidos Mexicanos, en su artículo 17, donde se establece que es un estudio sin riesgo para el paciente.

## RESULTADOS

Una vez realizada la aplicación del IASF, se evaluó un total de 263 alumnos, con una media de edad de 21,32 ± 1,89, donde el 66% fueron mujeres y el 34% correspondió a los hombres. 

Del total de ítems que contemplan el IASF, solo los ítems 4, 7, 8, 9, y 10 tuvieron porcentaje mayor al 15%, en la respuesta “Sí”, y se tomaron para los siguientes análisis (Tabla 1). 

**Tabla 1:** Distribución general de las respuestas del índice anamnésico simplificado de Fonseca (IASF) en la población estudiantil analizada

IASF	Respuestas			Sumatoria de respuestas
Ítems	No	A veces	Sí	(A veces + Sí)
	n (%)	n (%)	n (%)	n = %
1	244 (92,7)	17 (6,5)	2 (0,8)	19 (7)
2	208 (79,1)	50 (19)	5 (1,9)	55 (21)
3	182 (69,2)	71 (27)	10 (3,8)	81 (31)
4	157 (59,7)	57 (21,7)	49 (18,6)	106 (40)
5	189 (71,9)	62 (23,6)	12 (4,6)	74 (28)
6	250 (95,1)	13 (4,9)	0 (0)	13 (5)
7	141 (53,6)	69 (26,2)	53 (20,2)	122 (46)
8	162 (61,6)	51 (19,4)	50 (19)	101 (38)
9	138 (52,5)	79 (30)	46 (17,5)	125 (48)
10	116 (44,1)	70 (26,6)	77 (29,3)	147 (56)

La frecuencia con la que se presentó la TTM, distribuida por su severidad, en la población general de estudio se observa en la Tabla 2. La prevalencia total de los TTM en la población estudiantil fue del 63%; sin embargo, la manifestación leve fue la más frecuente, con un 44%.

**Tabla 2:** Distribución de la severidad de trastornos temporomandibulares utilizando el índice anamnésico simplificado de Fonseca (IASF) en la población estudiantil analizada

IASF	n(%)
No presenta	96 (37)
Leve	116 (44)
Moderado	43 (16)
Severo	8 (3)
Total	263 (100)

Las variables de interés fueron designadas como presencia de TTM: ítem 4: dolores de cabeza frecuentes; ítem 7: ruido en mandíbula cuando abres la boca, ítem 8: aprietas o rechinas los dientes, ítem 9: al cerrar la boca los dientes encajan mal, ítem 10: te consideras una persona nerviosa, y el sexo de los individuos, para el que se aplicó la prueba de independencia de Ji^2^. Los valores se muestran en la Tabla 3. 

**Tabla 3:** Distribución de los trastornos temporomandi-bulares, cefaleas, ruidos articulares, rechinido de dientes, mala oclusión, y nerviosismo, según el sexo, en la población de estudio

	Sexo		P Valor†
	Mujer	Hombre		
	n (%)	n (%)		
TTM	173 (66)	90 (34)		
			
No presenta	44 (25)	52 (57)	0,001
Leve	83 (48)	33 (37)	
Moderado	38 (22)	5 (6)	
Severo	8 (5)	0 (0)	
Ítem 4			
No	82 (47)	75 (83)	0,001
A veces	48 (28)	9 (10)	
Sí	43 (25)	6 (7)	
Ítem 7			
No	79 ( 46)	62 (69)	<0,001
A veces	48 ( 28)	21 ( 23)	
Sí	47 ( 27)	7 (8)	
Ítem 8			
No	92 (53)	60 (67)	0,021
A veces	40 (23)	21 (23)	
Sí	41 (24)	9 (10)	
Ítem 9			
No	75 (43)	63 (70)	<0,001
A veces	59 (34)	20 (22)	
Sí	39 (23)	7 (8)	
Ítem 10			
No	62 (36)	54 (60)	0,001
A veces	43 (25)	27 (30)	
Sí	68 (39)	9 (10)	

TTM: Trastornos temporomandibulares; † Prueba de Ji2 a una significancia del 95%. Ítem 4: Dolores de cabeza frecuentes; ítem 7: ruido en mandíbula cuando abres la boca; ítem 8: aprietas o rechinas los dientes; ítem 9: al cerrar la boca los dientes encajan mal; ítem 10: Te consideras una persona nerviosa.

**Figura 1:**
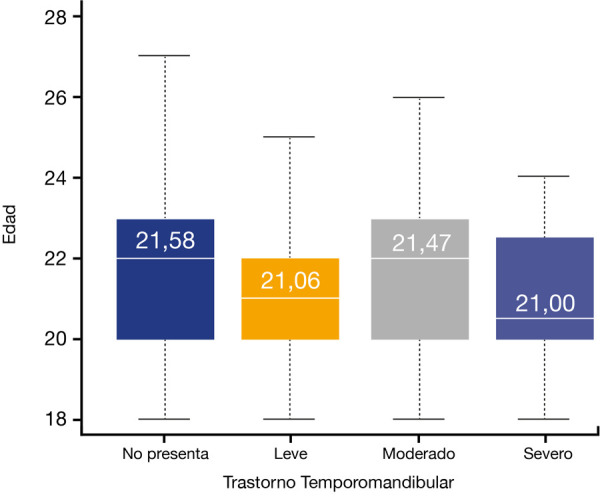
Diagrama de cajas que muestra las diferentes medias de edad respecto de la manifestación de las categorías de TTM en la población estudiada (prueba Anova de un factor, p = 0,211).

Para determinar si existían diferencias entre las medias de edades de las diferentes manifestaciones de TTM, se aplicó la prueba de Anova de un factor, la cual no mostró diferencias estadísticamente significativas (p = 0,211) (Figura 1).

Por último, el total de individuos que fueron positivos al IASF y respondieron como “A veces” y “Sí” de manera conjunta a los ítems de interés se muestran en la Tabla 4. 

**Tabla 4:** Individuos con respuestas positivas al IASF, de manera conjunta, a dolores de cabeza frecuentes, ruido en mandíbula cuando abres la boca, apretar o rechinar los dientes, al cerrar la boca los dientes encajan mal, y considerarse una persona nerviosa

	Sexo		
Ítems de interés presentes de manera conjunta	Mujeres	Hombres	Total
	n (%)	n (%)	n (%)
Ítem 4, ítem 7, ítem 8, ítem 9, ítem 10	25 (93%)	2 (7%)	27 (100%)

Ítem 4: Dolores de cabeza frecuentes; Ítem 7: Ruido en mandíbula cuando abres la boca; Ítem 8: Aprietas o rechinas los dientes; Ítem 9: Al cerrar la boca los dientes encajan mal; Ítem 10: Te consideras una persona nerviosa.

## DISCUSIÓN

La importancia de utilizar índices simplificados en el estudio de TTM radica en su fácil aplicación a gran escala, lo que permite un enfoque epidemiológico ^11^; así se logró determinar el comportamiento de los TTM en la evaluación de la población estudiantil de la Facultad de Odontología de la Universidad Juárez del Estado de Durango.

Es importante indicar que los TTM son afecciones de etiología multifactorial cuya prevalencia varía considerablemente entre poblaciones alrededor del mundo ^15^^-^^17^. Entre los hallazgos de este estudio, se hace notar la alta afectación por TTM entre los estudiantes, pues se aprecia generalmente una prevalencia del 63%; asimismo, la manifestación leve representa el 44%. Esto difiere con lo reportado por Loster *et al.*^18^, quienes mencionan una prevalencia del 26,5% en una población polaca, resultados que se obtuvieron mediante la implementación de los criterios diagnósticos de investigación para TTM (CDI/TTM).

Al comparar nuestros resultados epidemiológicos con otros estudios realizados en poblaciones estudiantiles, se observa de igual manera una ligera discrepancia, como lo muestra el estudio realizado por Acharya *et al.*^19^ en universitarios cursantes de medicina y odontología de Nepal, que halló una prevalencia de TTM en su manifestación leve del 41.2%. Algo similar se observa en la investigación de Habib *et al*. ^20^, quienes indican una prevalencia del 36.1% de TTM leves, y la efectuada por Zwiri y Al-Omiri ^3^ mediante la aplicación de un cuestionario con elementos relacionados con diferentes síntomas de TTM y posibles factores de riesgo, que reportó un 49,7% de prevalencia en universitarios de Arabia Saudita. Por otra parte, la prevalencia de TTM severo tuvo un bajo impacto general de tan solo el 3%, cifra que difiere de lo reportado por Guerrero *et al.*^7^, la cual fue del 19,6 %. Cabe mencionar que en este último no se utilizó el IASF como instrumento para determinar la presencia de TTM, y sí los CDI/TTM. 

La variación en la prevalencia de los TTM con respecto al sexo tiene relación con diversos factores como los emocionales, psicológicos, económicos y sociales ^21^. Guerrero *et al*. ^7^ reportan una prevalencia de TTM severos en mujeres del 28%, mientras que Pimenta *et al.*^22^ indican una mayor proporción de afectación en mujeres: 4/6 mujeres por cada hombre. Nuestros resultados arrojaron la presencia de TTM severo solo en mujeres, con un 3% de la población total, y en general indican una diferencia estadísticamente significativa entre hombres y mujeres con relación a la presencia de TTM leves, moderados y severos (p = 0,001).

Las alteraciones y afectaciones por TTM tienen relación con los cambios propios de la edad, pues se atribuye una mayor presencia de TTM en edades entre los 30-35 y 50-55 años, como picos máximos ^23^. Lo anterior contrasta con nuestros resultados, dadas las características de la población.

Dado que la etiología de los TTM es multifactorial ^3^, se torna complejo tomar signos y síntomas de manera individual puesto que, en la mayoría de los casos, estos desórdenes están acompañados por dolor, limitación en los movimientos de apertura, estrés, bruxismo y cefalea, por mencionar algunos factores importantes; sin embargo, es crucial identificar su presencia y resaltar su importancia en el desarrollo de los TTM.

Actualmente, la identificación de TTM mediante el uso del IASF no es un sinónimo de diagnóstico de la enfermedad -pues para ello es necesario un examen físico riguroso, historial médico y la implementación de estudios de imagen, en conjunto con síntomas y signos ^4^-, pero sí de un trastorno amplio que engloba un grupo de desórdenes, signos y síntomas de severidad variable que resultan en la mayor o menor limitación de las funciones normales de la ATM ^(1, 24)^.

Uno de los síntomas más incapacitantes que se pueden presentar en alguna enfermedad, síndrome o afección es la cefalea. Cerca del 96% de la población adulta ha sido afectada por esta condición a lo largo de su vida ^25^. Algunos autores señalan la íntima relación entre el dolor facial, la cefalea irradiada y los TTM, y aunque son entidades que se diagnostican de manera individual, la presencia de ambos en una persona es muy común, pues la presencia de una puede potencializar a la otra ^2^^,^^4^^,^^16^. El 40% de los individuos analizados refirió síntomas de cefalea y el 53% de las mujeres indicó que padecía esta condición. 

La relación entre ruidos articulares y TTM es muy estrecha, con una prevalencia de hasta el 17,6% ^26^ en adolescentes, lo que sugiere una disfunción de las estructuras que complementan la ATM. En la población analizada, la presencia de ruidos articulares representó, en total, el 46%; en cuanto a la diferencia entre sexo, fue del 27% y 8% en mujeres y hombres, respectivamente.

Otro factor importante que se presenta en los TTM es el apretamiento o rechinido dental, conocido conceptualmente como bruxismo, sobre el cual, actualmente, existe un debate acerca de si se trata de un desorden, un hábito o un comportamiento. Así mismo, es difícil catalogarlo como un desorden perjudicial establecido o un factor de riesgo para el desarrollo de problemas bucales ^27^. La literatura indica que existe una relación entre el bruxismo y los TTM, por lo cual se torna importante determinar su prevalencia de forma conjunta ^(9, 28)^. La prevalencia de esta afección en mujeres fue del 24% y de solo el 10% en hombres; para el total de la muestra fue del 38%.

La maloclusión dental juega un papel importante en la fisiología de la ATM, pues se trata de un factor establecido de disrupción articular; además, guarda cierta relación con la función masticatoria, la deglución y la respiración ^29^. Los resultados de este estudio indican que el 48% de la población analizada tiene la percepción de una maloclusión dental, apreciación que se presentó en el 30% de las mujeres y el 17,5% de los hombres. Se requieren evaluaciones dirigidas a determinar si en realidad existe una maloclusión dental y corroborar su correlación con la presencia de TTM.

Por su parte, el estrés se establece actualmente como un desencadenante de dolor en TTM y se sugiere tomarlo en cuenta al establecer estrategias de intervención y tratamiento. La literatura indica una alta prevalencia de estrés en individuos que padecen de TTM sometidos a estricto esfuerzo físico y mental ^30^. En lo que respecta a este estudio y de acuerdo con el ítem 10 del IASF, se encontró una respuesta positiva que podría indicar estrés en el 39% de las mujeres y el 10% de los hombres. En general, esta condición se presentó en el 56% del total de la muestra analizada; sin embargo, son necesarios más estudios que permitan establecer la etiología y prevalencia del estrés en nuestra población.

Con todo lo anterior, podríamos indicar que estos factores actúan como covariables y merecen un estudio más profundo a fin de establecer su etiología y relación etiológica con el desarrollo de TTM. Además, es relevante mencionar que la manifestación leve de TTM fue la más prevalente, con un 44%, casi la mitad de la población analizada. Al tratarse de una población joven, resulta importante ofrecer una orientación oportuna, preventiva y eficaz que permita minimizar factores desencadenantes, indicar cuidados o direccionar posibles terapias con el fin de evitar una evolución de estos trastornos que incremente su severidad.

Entre las limitaciones de este estudio, podemos mencionar que solo se aplicó el IASF y no se tuvo un instrumento que fungiera como *gold standard*; además, solo se recopilaron las respuestas de los ítems, algo que resulta ser condicionado por la autopercepción de los individuos ante un mismo problema. Si bien el propósito de este estudio fue estimar la prevalencia de TTM en una población estudiantil, se aconseja para futuros estudios el contar con un examen clínico bucal y articular a fin de poder corroborar los resultados de los instrumentos para la evaluación de TTM. Como perspectiva, se contempla añadir un estudio de imagen sobre las estructuras que conforman la ATM en aquellos individuos que resulten con una evaluación de TTM severo y con signos y síntomas indicativos de atención inmediata, a fin de comparar y correlacionar los resultados de las diferentes pruebas.

## CONCLUSIONES

Existe una alta prevalencia de TTM, cuya manifestación leve afecta con mayor frecuencia a la población estudiantil de la Universidad Juárez del Estado de Durango (México), y el sexo femenino es el que tiene una mayor presencia y manifestación de estos trastornos. Lo anterior es de vital importancia para trazar acciones preventivas y terapéuticas en la población estudiantil, sobre todo porque deben fungir como un reflejo de la atención que debe prestarse a la población en general. Además, estos hallazgos apoyarán la implementación de más estudios sobre esta problemática, que resulta tan compleja y multifactorial.

## Fuente de financiamiento:

Autofinanciado

## ANEXO 1 ANAMNESIS DE FONSECA

(Versión en español)

Edad: _________ Sexo: Masculino ___ Femenino___

El cuestionario está compuesto por diez preguntas para las cuales son posibles las respuestas: "sí"(10 puntos), "a veces" (5 puntos) y "no" (0 puntos).

Para cada pregunta, usted debe señalar solamente una respuesta.

1. ¿Es difícil para usted abrir la boca?

Sí___ No___ A veces___

2. ¿Se le dificulta mover la mandíbula hacia los lados?

Sí___ No___ A veces___

3. ¿Siente cansancio o dolor cuando mastica?

Sí___ No___ A veces___

4. ¿Tiene dolores de cabeza frecuentes?

Sí___ No___ A veces___

5. ¿Tiene dolor en la nuca o el cuello?

Sí___ No___ A veces___

6. ¿Tiene dolor de oído continuamente?

Sí___ No___ A veces___

7. ¿Siente ruido en la mandíbula cuando mastica o abre la boca?

Sí___ No___ A veces___

8. ¿Siente que aprieta o rechina (frota) los dientes?

Sí___ No___ A veces___

9. ¿Siente que al cerrar la boca sus dientes encajan mal?

Sí___ No___ A veces___

10. ¿Se considera una persona nerviosa (con estrés)?

Sí___ No___ A veces___

**Table:**

Puntaje total	Clase de disfunción temporomandibular
0 a 15 puntos	No presenta signos y síntomas
20 a 40 puntos	Leve
45 a 65 puntos	Moderado
70 a 100 puntos	Severo

Diagnóstico:
